# A preliminary composite of blood-based biomarkers to distinguish major depressive disorder and bipolar disorder in adolescents and adults

**DOI:** 10.1186/s12888-023-05204-x

**Published:** 2023-10-16

**Authors:** Jieping Huang, Xuejiao Hou, Moyan Li, Yingshuang Xue, Jiangfei An, Shenglin Wen, Zi Wang, Minfeng Cheng, Jihui Yue

**Affiliations:** 1 Department of Psychiatry, The Fifth Affiliated Hospital of Sun Yat-sen University, Zhuhai, 519000 China; 2 Zhuhai Promotion Association of Mental Health, Zhuhai, 519000 China

**Keywords:** Major depressive disorder, Bipolar disorder, Diagnosis, Nomogram, Validation

## Abstract

**Background:**

Since diagnosis of mood disorder heavily depends on signs and symptoms, emerging researches have been studying biomarkers with the attempt to improve diagnostic accuracy, but none of the findings have been broadly accepted. The purpose of the present study was to construct a preliminary diagnostic model to distinguish major depressive disorder (MDD) and bipolar disorder (BD) using potential commonly tested blood biomarkers.

**Methods:**

Information of 721 inpatients with an ICD-10 diagnosis of MDD or BD were collected from the electronic medical record system. Variables in the nomogram were selected by best subset selection method after a prior univariable screening, and then constructed using logistic regression with inclusion of the psychotropic medication use. The discrimination, calibration and internal validation of the nomogram were evaluated by the receiver operating characteristic curve (ROC), the calibration curve, cross validation and subset validation method.

**Results:**

The nomogram consisted of five variables, including age, eosinophil count, plasma concentrations of prolactin, total cholesterol, and low-density lipoprotein cholesterol. The model could discriminate between MDD and BD with an area under the ROC curve (AUC) of 0.858, with a sensitivity of 0.716 and a specificity of 0.890.

**Conclusion:**

The comprehensive nomogram constructed by the present study can be convenient to distinguish MDD and BD since the incorporating variables were common indicators in clinical practice. It could help avoid misdiagnoses and improve prognosis of the patients.

**Supplementary Information:**

The online version contains supplementary material available at 10.1186/s12888-023-05204-x.

## Background

Both major depressive disorder (MDD) and bipolar disorder (BD) are common chronic psychiatric disorders, which usually cause health damage or even severe disability to patients [1]. Accurate diagnosis of MDD and BD, which primarily depends on clinical manifestations, is the basis of individualized treatment and improvement of prognosis. However, the onset of BD is usually dominated by a depressive episode [2] and the clinical features of MDD and BD often overlap, which causes troubles to clinicians in diagnosis. And reducing misdiagnosis is critical to avoid delays of proper therapy or poorer outcomes.

Although BD is a multifactorial disorder with several subtypes, such as bipolar disorder I (BD I) and bipolar disorder II (BD II), some large genome-wide association studies have found that no significant locus identified for BD overlapped with those identified for depression, while all BD subtypes have common variant heritability [3]. This provides a theoretical basis for the potential existence of biomarkers to distinguish between MDD and BD. Emerging studies have shown that some potential indicators may help improve diagnostic accuracy between MDD and certain types of BD, or discriminate different phases of BD, such as biomarkers of individual system including the blood system [4] and the immune system [5],or biomarkers of multiple systems such as inflammation-immune response traits [6, 7], metabolic syndrome components [8, 9], or composites of potential gene or protein biomarkers via laboratory researches [10, 11].

Given the complexity and heterogeneity of etiology of mental diseases, biomarkers of multiple systems are more likely to effectively differentiate between MDD and BD. A composite of indicators from routinely measured examinations can not only reveal functions or status of different systems, but also have the potential to apply to clinical use given its easy accessibility.

In this study, we hypothesized that a panel of biomarkers combining routinely measured indicators might help differentiate between MDD and BD. In accordance, the aim of the study was to construct a preliminary prediction model to distinguish MDD and BD and evaluate its performance.

## Methods

### Study population

The study population consisted patients admitted to the Fifth Affiliated Hospital of Sun Yat-sen University from January 2019 to December 2021. For cases of repeated hospitalizations, only the first admission was included. Information on qualified cases was extracted from the electronic medical record system after an ethical review by the Ethic Committee of the hospital. As a retrospective clinical study, the requirement for informed consent was exempted and identifiable personal information was removed to protect patient privacy.

### Inclusion and exclusion criteria

The study included patients with an ICD-10 diagnosis of bipolar disorders (ICD-code: F31) or depressive disorders (ICD-code: F32 & F33) [12]. Although the ICD-codes include many subcategories that generally depend on the present clinical manifestation, we just used generic diagnosis of MDD and BD. In short, BD was diagnosed if the patient was having either (hypo)mania episode or depressive episode or having mixed or alternating (hypo)mania or depressive symptoms at survey, and had at least one episode of other mood disorder in the past. MDD was diagnosed if the patient was having depressive episode at survey but never had (hypo)mania episodes in the past. In order to avoid possible misdiagnosis, the diagnosis was cross checked by attending physicians, and finally confirmed by the Department Chief. Exclusion criteria were pregnancy, chronic infectious diseases including viral hepatitis and syphilis, autoimmune diseases including hashimoto thyroiditis and asthma, diabetes, malignant tumors or cancers. Therefore, a total of 721 participants were included in the study (supplement Fig. 1).

### Data collection

We collected epidemiological data of the 721 participants, including age, gender, duration of the diseases, and marital status. And we also recorded the use of several psychotropic drugs, including antipsychotics, antidepressants, mood stabilizers, and benzodiazepines, in the month preceding the study entry, which we marked as present psychotropic medication use. In addition, results of routine blood tests were also collected. The selected blood tests were performed in the morning on the second hospitalization day, from a forearm vein after at least 10 h of fasting.

### Potential predictors selection

Potential predictor selection was primarily performed using traditional statistical methods and machine learning approaches. Firstly, an initial variable screening was performed using univariable analysis [13], and only covariates with a *p*-value of less than 0.01 were chosen for subsequent analysis. Secondly, predictors were further selected from the above variables using the best subset selection method, via the leaps package (Version 3.1) with complete cases [14]. During the process, we set the maximum size of the subset to eight, which was also the default number in the function. During repeated iterations, information criterions including Mallows’ Cp (CP), and Bayes Information Criteria (BIC) of different subset sizes were demonstrated in plots, all of which help determining the best subset [15]. Thirdly, decision curve analysis (DCA) was used to choose the final model when different information criterion directed to different best subsets [16].

### Development of the preliminary prediction model

The preliminary prediction model was developed using logistic regression, and variables were excluded if their coefficients became insignificant after adjusting for the psychotropic medication use. Since variables were selected without considering observations with missing data, we used multiple imputation by chained equations (MICE) to avoid bias or inefficient estimates of parameters [17]. All results of the blood tests, in addition to age and gender, as well as the dichotomous outcome variable were included in the imputation. With the assumption that data were missing at random (MAR), the predictive mean matching (PMM) method was used to impute the missing variables using the mice package (version 3.15.0) in R. Since complete case analysis may introduce bias, we used imputed datasets for consistency checks. If the conclusions drawn from the observatory cases or imputed cases were consistent, we could be confident that the conclusions were reliable.

### Model presentation and examination

The preliminary prediction model was presented in the form of a nomogram and its performance, which was assessed in two aspects, discrimination and calibration, was examined using observatory data containing cases without missing values of the selected variables.

Discrimination refers to the ability to distinguish between the two outcomes and can be assessed by concordance statistic (c-statistic). In logistic regression analysis, the c-statistic is equal to the area under the ROC curve (AUC) [10]. The AUC with a higher value indicated higher accuracy. The model would be considered superior to a random ordering model if AUC > 0.5, while AUC value ranging from 0.5 to 0.7 indicate mild performance, 0.7–0.9 indicate moderate performance. In addition, sensitivity, specificity, and the likelihood ratios [18], including the positive likelihood ratio (LR [+]) and the negative likelihood ratio (LR [-]) were calculated to further test the accuracy of the model .

Calibration is used to evaluate the goodness of fit of the prediction model, which was assessed by calibration curves [19], with the final regression model subjected to bootstrapping validation (1,000 bootstrap resamples), via the rms package (Version 6.3-0). In addition, the Hosmer-Lemeshow test was used for testing model fit.

Internal validation was performed using 10-fold cross-validation repeating 10 times [13], via caret package (Version 6.0–93). Moreover, different subsets were used to further validate the model, including the drug naïve subset, and the different age subgroups including 14–29 age group, 30–44 age group, and 45 + age group.

### Statistical analysis

Data for continuous variables are presented as mean and standard deviation (SD), skewed data as median (25th and 75th percentiles), and categorical variables as absolute numbers and percentages. Shapiro-Wilk test was used to check whether the continuous variables were normally distributed, and then Levene’s Test was used to assess the homogeneity of variance. Clinical characteristics were compared using Student’s t test for normally distributed variables of equal variance, or Welch T test for normally distributed variables without homogeneity of variance, or Wilcoxon rank sum test for skewed distributed variables, or Pearson’s Chi-squared test, Fisher’s exact test when required, for categorical variables. All statistical analyses were conducted using the freely available statistical software R (version 4.2.0). The reported statistical significance levels were all two-sided, with an alpha value set at 0.05.

## Results

### Epidemiological and clinical characteristics

In total, 721 patients were included in the current study, 234 in the MDD group and 487 in the BD group. Characteristics of the study population are given in Table 1. There were no statistically significant differences between patients with MDD and BD in gender, duration of illness, and family history of mental disorders, while patients of the two groups had different features in age, marital status, and use rate of antipsychotics, antidepressants, and mood stabilizers (*p* < 0.01).

**Table 1:** Epidemiological characteristics of patients with MDD or BP

Variables	Overall, N = 721	MDD, N = 234	BP, N = 487	*p*
Age, years	21 (16, 37)	47 (27, 57)	17 (15, 24)	< 0.001
Gender, No. (%)				0.061
Female	537 (74%)	164 (70%)	373 (77%)	
Male	184 (26%)	70 (30%)	114 (23%)	
Duration, years	2 (1, 4)	2 (0.5, 5)	2 (1, 4)	0.2
Family history, No. (%)	120 (17%)	38 (16%)	82 (17%)	0.8
Marital status, No. (%)				< 0.001
Divorced	20 (2.8%)	8 (3.4%)	12 (2.5%)	
Married	223 (31%)	150 (64%)	73 (15%)	
Single	474 (66%)	72 (31%)	402 (83%)	
Widowed	4 (0.6%)	4 (1.7%)	0 (0.0%)	
Antipsychotics use, No. (%)	332 (46%)	68 (29%)	264 (54%)	< 0.001
Antidepressants use, No. (%)	278 (39%)	124 (53%)	154 (32%)	< 0.001
Mood stabilizers use, No. (%)	277 (38%)	19 (8.1%)	258 (53%)	< 0.001
Benzodiazepines use, No. (%)	284 (39%)	101 (43%)	183 (38%)	0.2

Data are presented as median (IQR) or number (percentage). *P* value is derived from univariable analyses using Wilcoxon rank sum test or Pearson’s Chi-squared test (Fisher’s exact test when needed). The psychotropic medication uses were limited to a month preceding study entry

Notably, 226 (31.34%) participants had different degrees of data missing in the results of blood tests, most of which were concentrated on the examinations of the inflammatory and immune response (Supplement Fig. 2).

### Variable selection

**Fig. 1:**
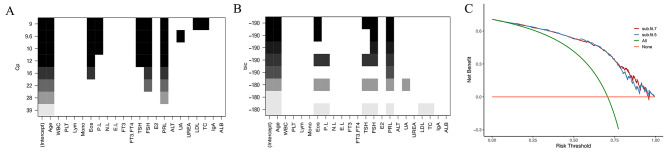
(**A**-**B**) Best models for each subset size based on Mallows’ Cp (CP) and Bayes Information Criteria (BIC). (**C**) Decision curve analysis for the model with 5 variables (sub.fit.5) and the model with 7 variables (sub.fit.7). *WBC, white blood cell count; PLT: platelet count; Lym, lymphocyte count; Eos, eosinophil count; P.L, platelet-to-lymphocyte ratio; N.L, neutrophil-to-lymphocyte ratio; E.L, eosinophil-to-lymphocyte ratio; FT3, free triiodothyronine; FT3.FT4, free triiodothyronine-to-free thyroxine ratio; TSH, thyroid stimulating hormone; FSH, follicle stimulating hormone; E2, estradiol; PRL, prolactin; ALT, alanine transaminase; UA, uric acid; LDL, low density lipoprotein cholesterol; TC, total cholesterol; IgA, immunoglobulin A; ALB, albumin*

With preliminary data screening using univariable analysis, 22 potential biomarkers including age with a *p*-value < 0.01 were selected for best subset selection (Supplement Table). As demonstrated (Fig. 1), the subset with eight variables showed the smallest CP (Fig. 1A), while the subset with five variables showed the smallest BIC (Fig. 1B). However, for the former model, the regression coefficient for platelet-to-lymphocyte ratio (P.L) was not significant (p ≅ 0.09), which was deleted from the model after verifying that its exclusion did not make a significant statistic difference. Subsequently, DCA clarified that the model with seven variables had moderately greater clinical benefits in general (Fig. 1C), which consisted of age (unit: years), eosinophil count (Eos, unit: 10^9^/L), plasma concentrations of thyroid-stimulating hormone (TSH, unit: uIU/mL), follicle-stimulating hormone (FSH, unit: mIU/mL), prolactin (PRL, unit: ng/mL), total cholesterol (TC, unit: mmol/L), and low-density lipoprotein cholesterol (LDL, unit: mmol/L).

Multiple logistic regression analysis incorporating the 7 selected variables was shown in Table 2. After adjusting for the present psychotropic medication use, the coefficients (β) and odd ratios (or exp(β)) of TSH and FSH became insignificant, which resulted in the deletion of the two variables. The imputed dataset showed consistent results as the complete dataset.

**Table 2:** Multivariable regression for diagnosis between MDD and BP in patients

Intercept and variables	Model of complete cases			Model of multiple imputations		
	β	Odds Ratio (95% CI)	*P*	β	Odds Ratio (95% CI)	*P*
Intercept	2.539		< 0.001	2.587		< 0.001
EOS	2.358	10.6 (1.69 to 75.17)	0.015	2.392	10.9 (1.66 to 72.23)	0.013
TSH	0.102	1.10 (0.99 to 1.26)	0.104	0.104	1.11 (0.98 to 1.25)	0.098
FSH	-0.014	0.99 (0.97 to 1.00)	0.068	-0.013	0.99 (0.97 to 1.00)	0.091
PRL	0.012	1.01 (1.00 to 1.02)	0.006	0.012	1.01 (1.00 to 1.02)	0.004
LDL	0.873	2.39 (1.29 to 4.54)	0.006	0.882	2.42 (1.30 to 4.49)	0.005
TC	-0.698	0.50 (0.30 to 0.82)	0.007	-0.711	0.49 (0.30 to 0.81)	0.005
age	-0.067	0.93 (0.92 to 0.95)	< 0.001	-0.072	0.93 (0.91 to 0.95)	< 0.001

### Presentation of the preliminary prediction model

**Fig. 2:**
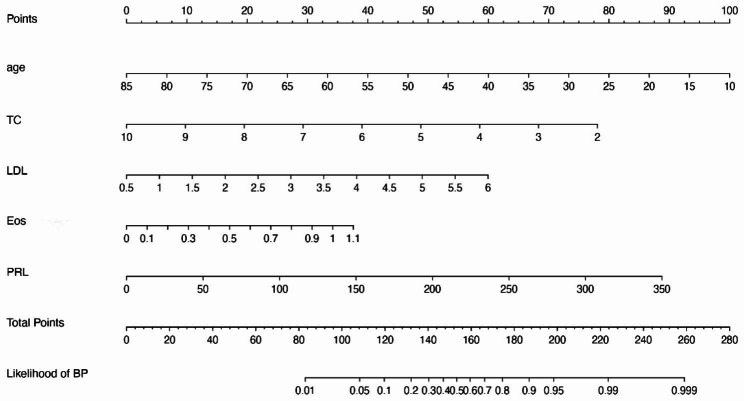
The nomogram developed in the observatory populations, incorporating age, total cholesterol (TC), and low-density lipoprotein cholesterol (LDL), eosinophil counts (Eos), and prolactin (PRL)

The final model incorporating the five potential independent predictors, age, LDL, TC, Eos, and PRL, was presented as a nomogram (Fig. 2).

### Evaluation of model performance

**Fig. 3:**
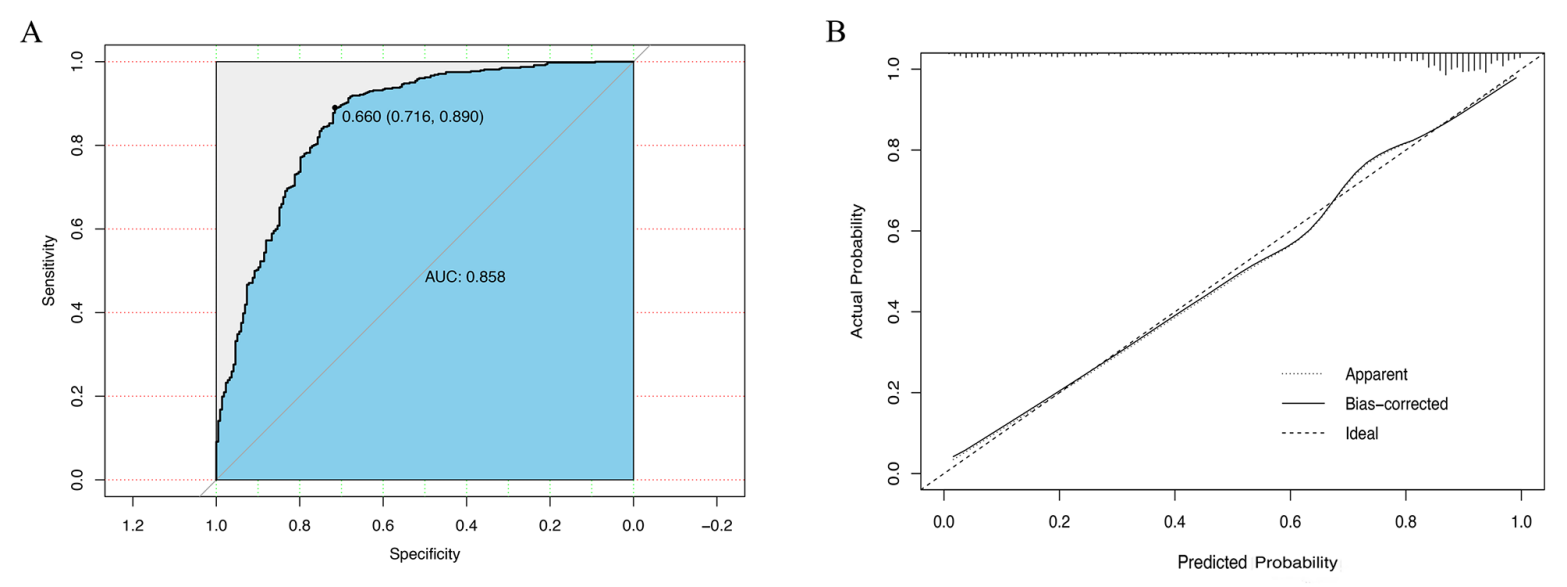
(**A**) Receiver operator characteristics (ROC) curve for the diagnostic model to distinguish patients with MDD or BD. For logistic regression models, c-statistic is equal to Area Under the ROC Curve (AUC). (**B**)Calibration curve. The x-axis represents the predicted probability and y-axis represents the actual probability of BD diagnosis. Perfect prediction would correspond to the 45° dashed line, the dotted line represents the observatory cases (n = 700), the solid line is bias-corrected by bootstrapping (B = 1000 repetitions)

For the above nomogram, the c-statistic was 0.858, indicating good discrimination (Fig. 3A). Moreover, with a cutoff value of 0.66, the model showed a sensitivity of 0.716 and a specificity of 0.890. Moreover, LR [+] and LR [-] were 6.51 and 0.32, suggesting moderate shifts in probability of a correct diagnosis using the model.

The calibration plot indicated that predicted probabilities approximately matched actual probabilities for this model (Fig. 3B). And the Hosmer-Lemeshow test *p*-value was 0.705, indicating good model fit.

### Validation of the preliminary prediction model

**Fig. 4:**
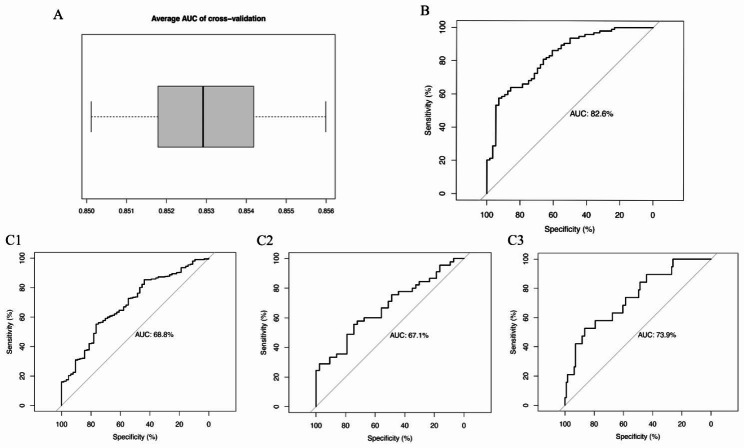
(**A**) Box plot showed the results of average AUC, or c- statistic, on the 100 cross-validation samples (10-fold cross validation repeated 10 times). (B, C1-C3) ROC curve distinguished patients with MDD or BD in different subgroups, including drug naïve group (B) and different age groups (C1-C3), 14–29 age group, 30–44 age group, and 45 + age group respectively

The average c-statistic of the repeated cross validation was 0.853 (range from 0.850 to 0.856) (Fig. 4A). This was close to but slightly lower than the overall model c-statistic of 0.858, indicating the stability and reliability of the preliminary predictions within the study population. Moreover, subset validation with ROC curve furtherly confirmed the robustness of the model. In the drug naïve subset, the AUC was 0.826, indicating good discrimination (Fig. 4B). In different age subsets, the AUC ranged from 0.671 to 0.739, indicating mild to moderate discrimination (Fig. 4C1-C3).

## Discussion

After many years of effort, researchers have not yet constructed a prediction model for discriminating between BD and MDD with clinical utility. In the present study, we preliminarily developed and validated a diagnostic nomogram, with a composite of biomarkers from routinely tested blood results, to distinguish MDD and BD. The model was constructed using the best subset selection method and then verified using multiple imputations and adjusted with the inclusion of the psychotropic medication use. The final model consisted of five variables: age, LDL, TC, Eos, and PRL. The model could discriminate between MDD and BD with an AUC of 0.858, with a sensitivity of 0.716 and a specificity of 0.890.

During the construction of the model, 47 features were reduced to 22 potential predictors at the first step by univariant analysis, then the best subset selection method was managed to select seven prominent markers. Of the 721 patients in the study, only 495 without missing data were used for the primary multivariable selections. Then 700 patients were used for adjustment and evaluation of the model after deleting cases with incomplete values of the prominent variables, which made the findings relatively more robust than constructing and validating the model using the same population. Moreover, repeated cross validations were subsequently used to verify the model when the training dataset and test dataset did not overlap, and subset validations were used to test the effectiveness of the model in drug naïve patients and patients of different age groups.

The findings of the present study were somewhat consistent with previous studies. For example, age is one of the most profound distinguishing factors between MDD and BD, as it had been broadly accepted that the onset age of MDD is generally later than that of BD [20, 21]. However, we wanted to see how the performance of the composite biomarkers would change if the effect of age was minimized. The study divided patients into three groups, 14–29 age group, 30–44 age group, and 45 + age group respectively. Within each group, age became insignificant different between MDD and BD patients (data not shown). Unsurprisingly, the model discrimination had varying degrees of deteriorations, and the AUC were 0.688, 0.671, and 0.739 respectively, indicating that the model still had mild to moderate diagnostic efficiency in patients of same age group.

Moreover, eosinophil could also help discriminate the two disorders, which was consistent with previous studies. For example, it has been demonstrated that eosinophil counts were reduced in MDD patients [22], while the increased eosinophil function could be found in the late-stage of BD [23].

In addition, the inclusion of PRL in the model, one of the hormones secreted by pituitary gland, suggested that pituitary function might play a role in differentiating MDD and BD. However, previous studies on the pituitary gland mainly focused on the gland volume changes in mental disorders and their association with hyperactivity in the Hypothalamic-Pituitary-Adrenal axis [24–26]. Other hormones provided by pituitary gland besides adrenocorticotropic hormone could also have potential effects on mental disorders. In this study, TSH and FSH were tested statistically significant but excluded after adjustment for psychotropic medication use, which was in accordance with clinical consensus that endocrine is greatly influenced during the drug treatment for affective disorders [27]. Interestingly, PRL remained in the model after medication adjustment. However, these findings require further confirmation in drug-free patients.

In addition, LDL and TC were also included in the final model. These findings did not contradict previous findings that abnormal lipid metabolism was more prevalent in MDD and BD patients compared to health controls [28, 29]. However, few studies have compared the differences in lipid profile distribution between MDD and BD. Our study showed that BD patients had relatively higher LDL levels, while MDD patients had higher TC levels. Although these findings indicated different lipid profiles in MDD and BD patients, but both were consistent with the findings that patients with severe mental illnesses had increased risks for cardiovascular diseases [30, 31].

Moreover, like endocrine functions, lipid metabolism is also seriously affected by some kinds of psychotropic drugs, especially antipsychotics and mood stabilizers, such as clozapine, olanzapine, and valproate [32], which can ultimately result in hyperlipidemia or even obesity. As it was demonstrated in Table 1, the proportion of BD patients using antipsychotics and mood stabilizers was significantly higher than that of MDD patients, however, the coefficients of TC and LDL in the regression model remained significant after the adjustment of medication use in this study, indicating that the pharmacological effect was not the only reason for the differences in the lipid levels between the two groups. In other words, abnormal lipid metabolism may underlie the mental disorders. However, since the cholesterol level can be greatly influenced by living habits, such as diet and physical activities [33], the significance could not be applied to populations with different lifestyles.

Emerging studies have confirmed the potential roles of inflammation or immune-based biomarkers as predictive biomarker panels to differentiate MDD and BD, usually including C-reactive protein (CRP), interleukins, and complement components [5, 34–36]. However, the above related potential biomarkers were surprisingly excluded during model development, which was inconsistent with previous findings. For example, Chang et al. demonstrated that baseline CRP could serve as a discrimination biomarker for MDD and bipolar II disorder in drug naïve patients (cutoff value: 621.6 ng/mL; AUC value: 0.816), and patients with baseline CRP greater than 621.6 ng/mL had 28.2 higher odds of bipolar II disorder [37]. However, in our study, CRP level showed no statistical difference between MDD and BD and was excluded at the first step. The possible reason might be treatment effects as indicated by Chang’s work itself: the difference of CRP level would become narrower between MDD and bipolar II disorder after treatment. Another possible reason may be bias from concentrated missing values on inflammation and immune factors; although the multiple imputations had indicated that the missingness of the selected variables in the model was at random, it may not represent the same missing pattern of the potential predictors in question [38].

There were several limitations to this study. Firstly, behavior characteristics and psychological assessments failed to be included in the analysis process. Secondly, BD patients were not specifically classified into different clinical phases including (hypo)manic or depressive phase, mixed episode of BD, or rapid cycling BD. Thirdly, the participants were included when they were at acute phase and blood examination were performed on the second day, the process were limited by clinical practice of the hospital, and the results may need further evaluation with participants in remission. At last, the data were collected from one hospital, the generalizability of the preliminary prediction panel needs further testing with external validation cohort.

Besides exploring the distributional differences of the blood indicators, emerging researches have been investigating the pathophysiology of MDD and BD in multiple molecular levels [39]. As the technology continuously develops and the cost deceases, it could be expected that a valid and convenient composite of biomarkers be constructed by combining biomolecular components and the ordinary clinical indictors, which could effectively distinguish between MDD and BD and also guide precision treatment in the future.

## Conclusion

Our study presents a nomogram that incorporates factors from commonly tested blood indicators that could conveniently help distinguishing MDD and BD, and thus reduce misdiagnosis.

### Electronic supplementary material

Below is the link to the electronic supplementary material.

Supplementary Material 1: Supplement table: Distribution of blood indicators of complete cases

Supplementary Material 2: Supplement figure 1: Flow chart of participants' enrollment process

Supplementary Material 3: Supplement figure 2: Missing patterns of the variables

## Data Availability

The data of this study cannot be made publicly available for confidentiality reasons. Data are however available from the corresponding author upon reasonable request.

## Abbreviations

MDD: Major depressive disorder
BD: Bipolar disorder
ROC: the receiver operating characteristic curve
AUC: area under the ROC curve
CP: Mallows’ Cp
BIC: Bayes Information Criteria
DCA: decision curve analysis
MICE: multiple imputation by chained equations
MAR: Missing at random
PMM: predictive mean matching
c-statistic: concordance statistic
LR [+]: positive likelihood ratio
LR [-]: negative likelihood ratio
SD: standard deviation
Eos: eosinophil count
TSH: thyroid-stimulating hormone
FSH: follicle-stimulating hormone
PRL: prolactin
TC: total cholesterol
LDL: low-density lipoprotein cholesterol
CRP: C-reactive protein

## References

[1] Kalin NH. New Insights into Major Depression and the treatment of Bipolar Depression, (in eng). Am J Psychiatry. Dec 2021;178(12):1071–4. 10.1176/appi.ajp.2021.21101042. 10.1176/appi.ajp.2021.21101042 34855453

[2] Grande I, Berk M, Birmaher B, Vieta E. “Bipolar disorder,“ (in eng), *Lancet*, vol. 387, no. 10027, pp. 1561–1572, Apr 09 2016, 10.1016/S0140-6736(15)00241-X. 10.1016/S0140-6736(15)00241-X 26388529

[3] Stahl EA, et al. Genome-wide association study identifies 30 loci associated with bipolar disorder, (in eng). Nat Genet. May 2019;51(5):793–803. 10.1038/s41588-019-0397-8. 10.1038/s41588-019-0397-8 PMC6956732 31043756

[4] Fusar-Poli L et al. “Neutrophil-to-Lymphocyte, Platelet-to-Lymphocyte and Monocyte-to-Lymphocyte Ratio in Bipolar Disorder,“ (in eng), *Brain Sci*, vol. 11, no. 1, Jan 06 2021, 10.3390/brainsci11010058. 10.3390/brainsci11010058 PMC7825034 33418881

[5] Perry BI, Upthegrove R, Kappelmann N, Jones PB, Burgess S, Khandaker GM. Associations of immunological proteins/traits with schizophrenia, major depression and bipolar disorder: a bi-directional two-sample mendelian randomization study, (in eng). Brain Behav Immun. Oct 2021;97:176–85. 10.1016/j.bbi.2021.07.009. 10.1016/j.bbi.2021.07.009 PMC7612947 34280516

[6] Jones GH, Vecera CM, Pinjari OF, Machado-Vieira R. Inflammatory signaling mechanisms in bipolar disorder, (in eng). J Biomed Sci. Jun 11 2021;28(1):45. 10.1186/s12929-021-00742-6. 10.1186/s12929-021-00742-6 PMC8194019 34112182

[7] Ruiz NAL, et al. Inflammatory process and Immune System in Major Depressive Disorder, (in eng). Int J Neuropsychopharmacol. Jan 12 2022;25(1):46–53. 10.1093/ijnp/pyab072. 10.1093/ijnp/pyab072 PMC8756095 34724041

[8] Gimenez-Palomo A, et al. Does metabolic syndrome or its component factors alter the course of bipolar disorder? A systematic review. Neurosci Biobehav Rev. Jan 2022;132:142–53. 10.1016/j.neubiorev.2021.11.026. 10.1016/j.neubiorev.2021.11.026 34800584

[9] Chan KL, Cathomas F, Russo SJ. “Central and Peripheral Inflammation Link Metabolic Syndrome and Major Depressive Disorder,“ (in eng), *Physiology (Bethesda)*, vol. 34, no. 2, pp. 123–133, Mar 01 2019, 10.1152/physiol.00047.2018. 10.1152/physiol.00047.2018 PMC6586832 30724127

[10] Munkholm K, Vinberg M, Pedersen BK, Poulsen HE, Ekstrøm CT, Kessing LV. A multisystem composite biomarker as a preliminary diagnostic test in bipolar disorder, (in eng). Acta Psychiatr Scand. Mar 2019;139(3):227–36. 10.1111/acps.12983. 10.1111/acps.12983 30383306

[11] Rhee SJ et al. “Comparison of serum protein profiles between major depressive disorder and bipolar disorder,“ (in eng), *BMC Psychiatry*, vol. 20, no. 1, p. 145, Apr 03 2020, 10.1186/s12888-020-02540-0. 10.1186/s12888-020-02540-0 PMC7118970 32245436

[12] W. H. Organization(WHO), *The ICD-10 classification of mental and behavioural disorders*, 1993. 10.1007/BF00788743 8284737

[13] Au EH, Francis A, Bernier-Jean A, Teixeira-Pinto A. “Prediction modeling-part 1: regression modeling,“ (in eng), *Kidney Int*, vol. 97, no. 5, pp. 877–884, May 2020, 10.1016/j.kint.2020.02.007. 10.1016/j.kint.2020.02.007 32247633

[14] Heinze G, Wallisch C, Dunkler D. Variable selection - A review and recommendations for the practicing statistician, (in eng). Biom J. May 2018;60(3):431–49. 10.1002/bimj.201700067. 10.1002/bimj.201700067 PMC5969114 29292533

[15] Kabacoff RI (2015). R in action.

[16] Fitzgerald M, Saville BR, Lewis RJ. “Decision curve analysis,“ (in eng), *JAMA*, vol. 313, no. 4, pp. 409 – 10, Jan 27 2015, 10.1001/jama.2015.37. 10.1001/jama.2015.37 25626037

[17] Blazek K, van Zwieten A, Saglimbene V, Teixeira-Pinto A. “A practical guide to multiple imputation of missing data in nephrology,“ (in eng), *Kidney Int*, vol. 99, no. 1, pp. 68–74, Jan 2021, 10.1016/j.kint.2020.07.035. 10.1016/j.kint.2020.07.035 32822702

[18] Weatherall M. “Information provided by diagnostic and screening tests: improving probabilities,“ (in eng), *Postgrad Med J*, vol. 94, no. 1110, pp. 230–235, Apr 2018, 10.1136/postgradmedj-2017-135273. 10.1136/postgradmedj-2017-135273 29133377

[19] Huang YQ, et al. Development and validation of a Radiomics Nomogram for Preoperative Prediction of Lymph Node Metastasis in Colorectal Cancer, (in eng). J Clin Oncol. Jun 20 2016;34(18):2157–64. 10.1200/JCO.2015.65.9128. 10.1200/JCO.2015.65.9128 27138577

[20] Vieta E et al. Early intervention in bipolar disorder, (in eng), Am J Psychiatry, vol. 175, no. 5, pp. 411–26, May 01 2018, 10.1176/appi.ajp.2017.17090972. 10.1176/appi.ajp.2017.17090972 29361850

[21] Park LT, Zarate CA. Depression in the primary care setting, (in eng), N Engl J Med, vol. 380, no. 6, pp. 559–68, Feb 07 2019, 10.1056/NEJMcp1712493. 10.1056/NEJMcp1712493 PMC6727965 30726688

[22] Singh D, et al. Changes in leukocytes and CRP in different stages of major depression, (in eng). J Neuroinflammation. Apr 04 2022;19(1):74. 10.1186/s12974-022-02429-7. 10.1186/s12974-022-02429-7 PMC8981816 35379263

[23] Panizzutti B et al. Increased serum levels of eotaxin/CCL11 in late-stage patients with bipolar disorder: an accelerated aging biomarker? (in eng), J Affect Disord, vol. 182, pp. 64 – 9, Aug 15 2015, 10.1016/j.jad.2014.12.010. 10.1016/j.jad.2014.12.010 25973785

[24] Delvecchio G, et al. Pituitary gland shrinkage in bipolar disorder: the role of gender, (in eng). Compr Psychiatry. Apr 2018;82:95–9. 10.1016/j.comppsych.2018.01.014. 10.1016/j.comppsych.2018.01.014 29454165

[25] Delvecchio G, Altamura AC, Soares JC, Brambilla P. “Pituitary gland in Bipolar Disorder and Major Depression: Evidence from structural MRI studies: Special Section on “Translational and Neuroscience Studies in Affective Disorders”. Section Editor, Maria Nobile MD, PhD. This Section of JAD focuses on the relevance of translational and neuroscience studies in providing a better understanding of the neural basis of affective disorders. The main aim is to briefly summarise relevant research findings in clinical neuroscience with particular regards to specific innovative topics in mood and anxiety disorders,“ (in eng), *J Affect Disord*, vol. 218, pp. 446–450, Aug 15 2017, 10.1016/j.jad.2017.03.066. 10.1016/j.jad.2017.03.066 28412090

[26] Keller J, et al. HPA axis in major depression: cortisol, clinical symptomatology and genetic variation predict cognition, (in eng). Mol Psychiatry. Apr 2017;22(4):527–36. 10.1038/mp.2016.120. 10.1038/mp.2016.120 PMC5313380 27528460

[27] Hayes JF, Marston L, Walters K, Geddes JR, King M, Osborn DP. Adverse renal, endocrine, hepatic, and metabolic events during maintenance Mood stabilizer treatment for bipolar disorder: a Population-Based Cohort Study, (in eng). PLoS Med. Aug 2016;13(8):e1002058. 10.1371/journal.pmed.1002058. 10.1371/journal.pmed.1002058 PMC4970809 27483368

[28] Wei YG, et al. Cholesterol and triglyceride levels in first-episode patients with major depressive disorder: a meta-analysis of case-control studies. J Affect Disord. Apr 1 2020;266:465–72. 10.1016/j.jad.2020.01.114. 10.1016/j.jad.2020.01.114 32056914

[29] Dalkner N et al. “Metabolic Syndrome Impairs Executive Function in Bipolar Disorder,“ (in eng), *Front Neurosci*, vol. 15, p. 717824, 2021, 10.3389/fnins.2021.717824. 10.3389/fnins.2021.717824 PMC8385126 34456679

[30] Goldstein BI et al. “Major Depressive Disorder and Bipolar Disorder Predispose Youth to Accelerated Atherosclerosis and Early Cardiovascular Disease: A Scientific Statement From the American Heart Association,“ (in eng), *Circulation*, vol. 132, no. 10, pp. 965 – 86, Sep 08 2015, 10.1161/CIR.0000000000000229. 10.1161/CIR.0000000000000229 26260736

[31] Nielsen RE, Banner J, Jensen SE. Cardiovascular disease in patients with severe mental illness, (in eng). Nat Rev Cardiol. Feb 2021;18(2):136–45. 10.1038/s41569-020-00463-7. 10.1038/s41569-020-00463-7 33128044

[32] Pillinger T et al. “Comparative effects of 18 antipsychotics on metabolic function in patients with schizophrenia, predictors of metabolic dysregulation, and association with psychopathology: a systematic review and network meta-analysis,“ (in eng), *Lancet Psychiatry*, vol. 7, no. 1, pp. 64–77, Jan 2020, 10.1016/S2215-0366(19)30416-X. 10.1016/S2215-0366(19)30416-X PMC7029416 31860457

[33] Bauer UE, Briss PA, Goodman RA, Bowman BA. “Prevention of chronic disease in the 21st century: elimination of the leading preventable causes of premature death and disability in the USA,“ (in eng), *Lancet*, vol. 384, no. 9937, pp. 45–52, Jul 05 2014, 10.1016/S0140-6736(14)60648-6. 10.1016/S0140-6736(14)60648-6 24996589

[34] Fries GR, Zamzow MJ, Andrews T, Pink O, Scaini G, Quevedo J. Accelerated aging in bipolar disorder: a comprehensive review of molecular findings and their clinical implications, (in eng). Neurosci Biobehav Rev. May 2020;112:107–16. 10.1016/j.neubiorev.2020.01.035. 10.1016/j.neubiorev.2020.01.035 32018037

[35] Haenisch F et al. “Towards a blood-based diagnostic panel for bipolar disorder,“ (in eng), *Brain Behav Immun*, vol. 52, pp. 49–57, Feb 2016, 10.1016/j.bbi.2015.10.001. 10.1016/j.bbi.2015.10.001 26441135

[36] Yu H et al. “Association of the plasma complement system with brain volume deficits in bipolar and major depressive disorders,“ (in eng), *Psychol Med*, pp. 1–11, Oct 26 2022, 10.1017/S0033291722003282. 10.1017/S0033291722003282 36285542

[37] Chang HH, et al. C-reactive protein: a differential biomarker for major depressive disorder and bipolar II disorder, (in eng). World J Biol Psychiatry. Feb 2017;18(1):63–70. 10.3109/15622975.2016.1155746. 10.3109/15622975.2016.1155746 26895280

[38] White IR, Royston P, Wood AM. “Multiple imputation using chained equations: Issues and guidance for practice,“ (in eng), *Stat Med*, vol. 30, no. 4, pp. 377 – 99, Feb 20 2011, 10.1002/sim.4067. 10.1002/sim.4067 21225900

[39] Mokhtari A, et al. The molecular pathophysiology of mood disorders: from the analysis of single molecular layers to multi-omic integration. Prog Neuropsychopharmacol Biol Psychiatry. Jun 8 2022;116:110520. 10.1016/j.pnpbp.2022.110520. 10.1016/j.pnpbp.2022.110520 35104608

