# Supplementary material for: A preliminary composite of blood-based biomarkers to distinguish major depressive disorder and bipolar disorder in adolescents and adults
Source: BMC Psychiatry. 2023 Oct 16;23:755. doi: 10.1186/s12888-023-05204-x (PMC10580619; doi:10.1186/s12888-023-05204-x)
Supplement: Supplementary file 1 — Supplementary Material 1: Supplement table: Distribution of blood indicators of complete cases [file 12888_2023_5204_MOESM1_ESM.docx]

Supplement table: Clinical characteristics of complete cases

|  |
| --- |
|  |

| Variables | MDD, N = 145^1^ | BP, N = 350^1^ | *P* |  |
| --- | --- | --- | --- | --- |
| WBC, 10^9^/L | 6.13 (4.69, 7.16) | 6.38 (5.41, 7.50) | 0.005 |  |
| PLT, 10^9^/L | 246 (206, 285) | 262 (218, 307) | 0.005 |  |
| Hb, g/L | 132 (122, 143) | 129 (120, 141) | 0.3 |  |
| NE, 10^9^/L | 3.31 (2.49, 4.24) | 3.31 (2.53, 4.18) | 0.6 |  |
| Lym, 10^9^/L | 1.92 (1.58, 2.38) | 2.33 (1.93, 2.70) | < 0.001 |  |
| Mono,10^9^/L | 0.40 (0.32, 0.49) | 0.43 (0.35, 0.55) | 0.006 |  |
| Eos, 10^9^/L | 0.11 (0.07, 0.19) | 0.18 (0.11, 0.27) | < 0.001 |  |
| BAS, 10^9^/L | 0.03 (0.02, 0.04) | 0.04 (0.02, 0.05) | 0.12 |  |
| P/L | 120(99, 156) | 113(92,138) | 0.005 |  |
| N/L | 1.65 (1.22, 2.19) | 1.41(1.07, 1.87) | 0.001 |  |
| M/L | 0.20(0.16, 0.26) | 0.19 (0.14, 0.24) | 0.039 |  |
| B/L | 0.02(0.010,0.02) | 0.02(0.01,0.02) | 0.13 |  |
| E/L | 0.06 (0.04, 0.09) | 0.07(0.05, 0.11) | < 0.001 |  |
| FT3, pmol/L | 4.37 (3.92, 4.89) | 4.67 (4.24, 5.15) | < 0.001 |  |
| FT4, pmol/L | 15.89(14.00, 18.03) | 15.27 (13.64, 17.25) | 0.064 |  |
| FT3/FT4 | 0.28(0.24, 0.32) | 0.31 (0.27,0.35) | < 0.001 |  |
| TSH, uIU/mL | 1.68 (1.09, 2.42) | 2.09 (1.37, 3.29) | < 0.001 |  |
| FSH, mIU/mL | 7 (4, 16) | 5 (3, 7) | < 0.001 |  |
| LH, mIU/mL | 7 (5, 19) | 7 (5, 13) | 0.4 |  |
| E2, pg/mL | 33 (19, 70) | 46 (31, 84) | < 0.001 |  |
| T, ng/mL | 0.39 (0.23, 3.53) | 0.35 (0.22, 1.81) | 0.4 |  |
| PRL, ng/mL | 22 (15, 34) | 33 (22, 58) | < 0.001 |  |
| RF, IU/mL | 6.58 (4.47, 7.81) | 6.43 (4.91, 7.26) | 0.4 |  |
| ASO, IU/mL | 30 (15, 76) | 39 (10, 106) | 0.3 |  |
| CRP, mg/L | 2.90 (1.80, 4.40) | 2.98 (1.66, 4.30) | 0.7 |  |
| AST, U/L | 17 (14, 21) | 15 (13, 19) | 0.011 |  |
| ALT, U/L | 13 (10, 20) | 10 (7, 16) | < 0.001 |  |
| UA, μmol/L | 308 (254, 365) | 332 (278, 399) | 0.001 |  |
| sCr, μmol/L | 68 (58, 76) | 64 (56, 74) | 0.13 |  |
| UREA, μmol/L | 4.00 (3.30, 5.00) | 3.62 (3.10, 4.40) | 0.001 |  |
| LDH, U/L | 153 (134, 173) | 157 (138, 182) | 0.11 |  |
| ApoE, mg/L | 43 (36, 50) | 41 (35, 49) | 0.15 |  |
| LDL, mmol/L | 2.39 (1.95, 3.01) | 2.20 (1.82, 2.67) | 0.006 |  |
| HDL, mmol/L | 1.23(1.01,1.50) | 1.22 (1.00,1.45) | > 0.9 |  |
| TC, mmol/L | 4.29 (3.79, 5.09) | 4.04 (3.49, 4.58) | < 0.001 |  |
| TG, mmol/L | 1.06 (0.78, 1.50) | 0.97 (0.72, 1.36) | 0.038 |  |
| C3, g/L | 1.07 (0.95, 1.24) | 1.10 (0.98, 1.22) | 0.3 |  |
| C4, g/L | 0.21 (0.17, 0.25) | 0.21 (0.17, 0.25) | 0.3 |  |
| IgM, g/L | 1.16 (0.82, 1.54) | 1.15 (0.88, 1.47) | 0.8 |  |
| IgA, g/L | 2.01 (1.58, 2.95) | 1.89 (1.41, 2.36) | < 0.001 |  |
| IgG, g/L | 12.27(10.98, 14.17) | 12.07 (10.59, 13.60) | 0.051 |  |
| ALB, g/L | 42.1 **± 3.36** | 43.4 **±** 3.14 | < 0.001 |  |
| GLOB, g/L | 24.7 (22.5, 26.9) | 24.2 (22.0, 26.4) | 0.14 |  |
| A/G | 1.73 (1.53, 1.84) | 1.79 (1.62, 1.97) | 0.001 |  |

Data are presented as mean **±** standard deviation (SD) or median (IQR). *P* value is derived from univariable analyses using student’s t test or Wilcoxon rank sum test.

*WBC, white blood cell count; PLT: platelet count; Hb, hemoglobin; Lym, lymphocyte count; Mono, monocyte count; Eos, eosinophil count; Bas, basophil count; P/L, platelet-to-lymphocyte ratio; N/L, neutrophil-to-lymphocyte ratio; M/L, monocyte -to-lymphocyte ratio; E/L, eosinophil-to-lymphocyte ratio; B/L: basophil -to-lymphocyte ratio; FT3, free triiodothyronine; FT4, free thyroxin; FT3/FT4, free triiodothyronine-to-free thyroxine ratio; TSH, thyroid stimulating hormone; FSH, follicle stimulating hormone; LH, luteinizing hormone; E2, estradiol; T, testosterone; PRL, prolactin; RF, rheumatoid factors; ASO, antistreptolysin; CRP, C-reactive protein; AST, aspartate transaminase; ALT, alanine transaminase; UA, uric acid; sCr: serum creatinine; UREA, urea; LDH, lactate dehydrogenase; ApoE, apolipoprotein E; LDL, low density lipoprotein cholesterol; HDL, high density lipoprotein cholesterol; TC, total cholesterol; TG, triglyceride; C3, complement C3; C4, complement C4; IgM, immunoglobulin M; IgA, immunoglobulin A; IgG, immunoglobulin G; ALB, albumin; GLOB, globulin; A/G, albumin-to-globulin ratio.*
