# Supplementary figures and images for: A preliminary composite of blood-based biomarkers to distinguish major depressive disorder and bipolar disorder in adolescents and adults
Source: BMC Psychiatry. 2023 Oct 16;23:755. doi: 10.1186/s12888-023-05204-x (PMC10580619; doi:10.1186/s12888-023-05204-x)

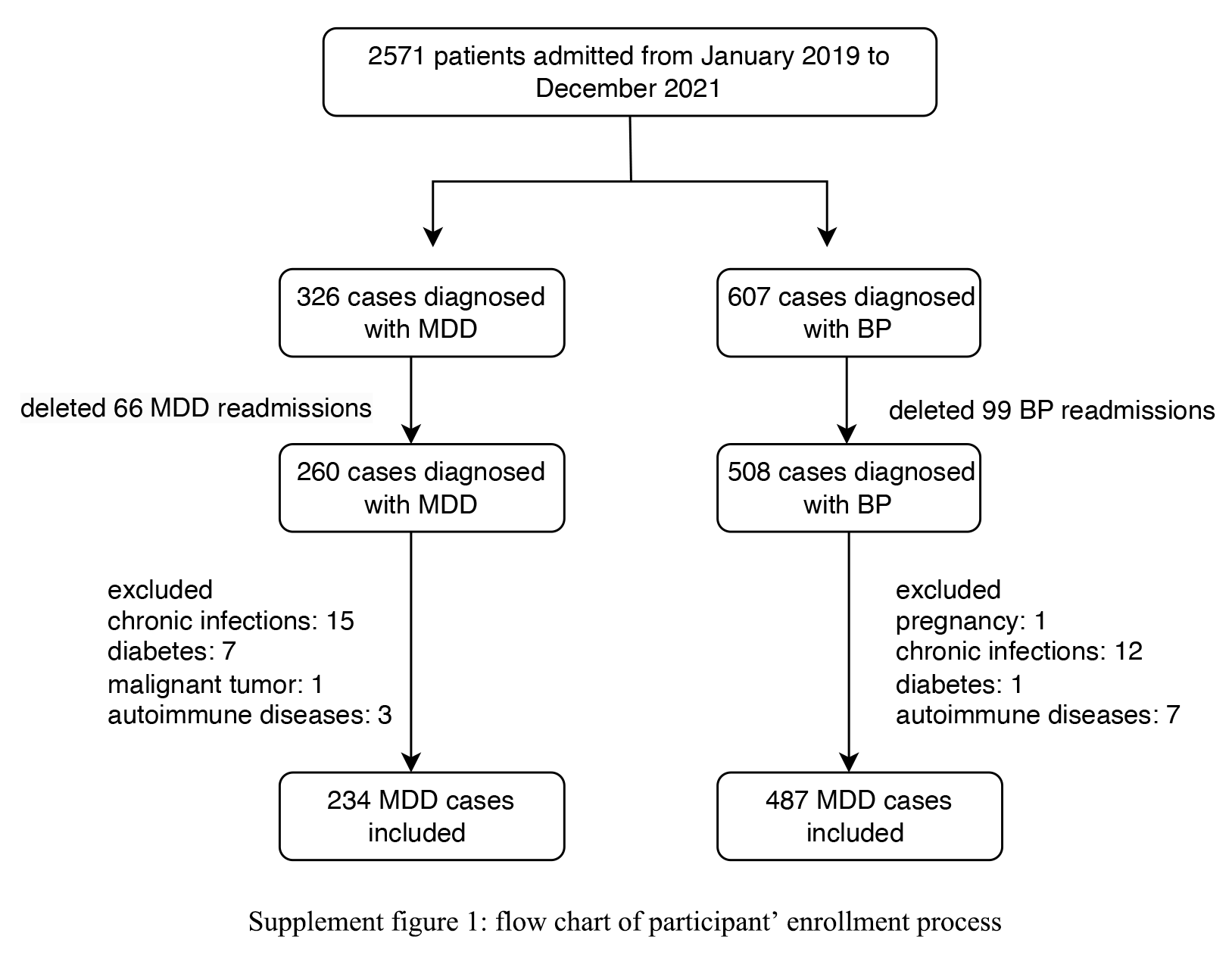

Supplement: Supplementary file 2 — Supplementary Material 2: Supplement figure 1: Flow chart of participants' enrollment process [file 12888_2023_5204_MOESM2_ESM.png]

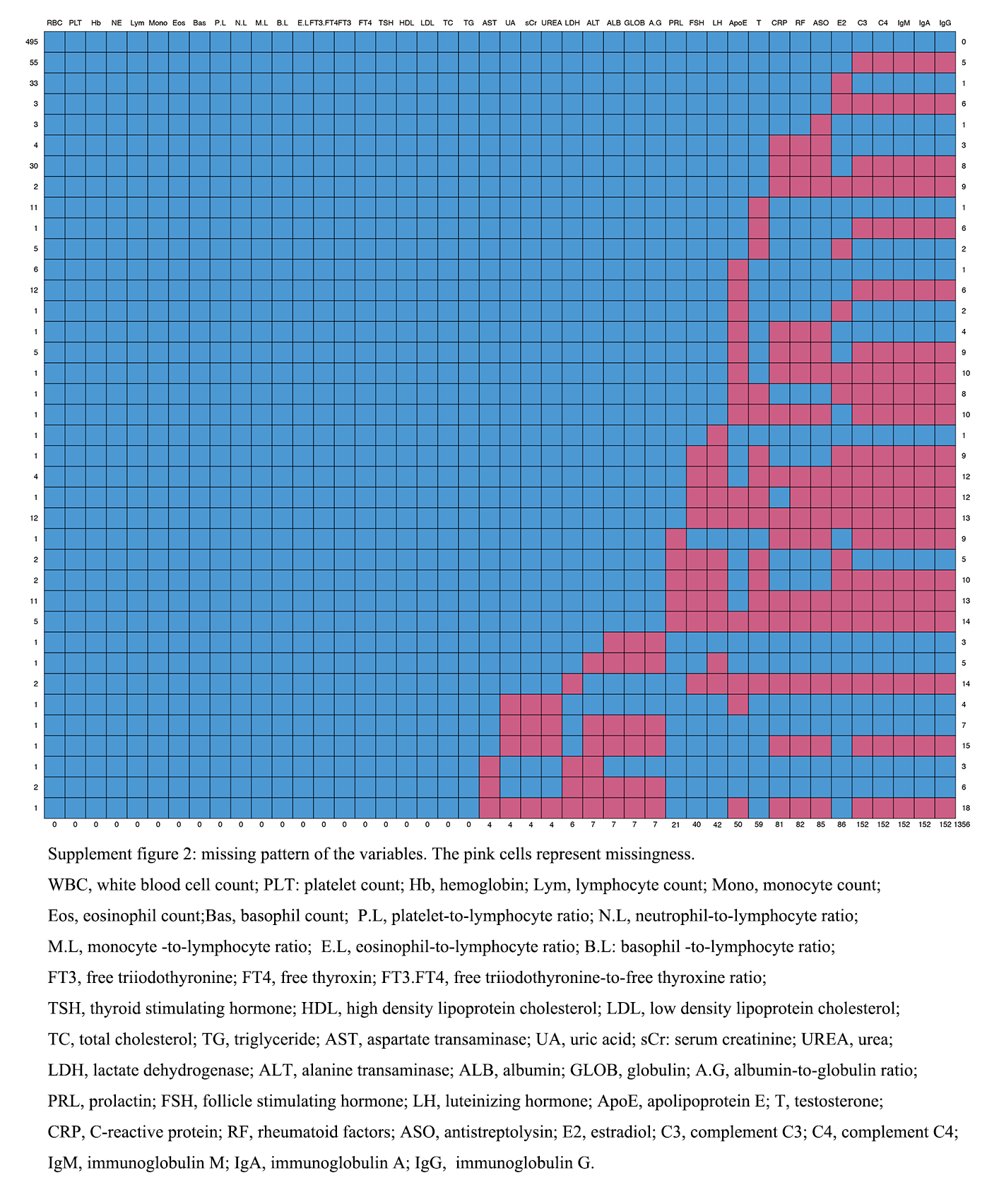

Supplement: Supplementary file 3 — Supplementary Material 3: Supplement figure 2: Missing patterns of the variables [file 12888_2023_5204_MOESM3_ESM.png]
